
JOURNAL INFORMATION
==============================
NLM Title Abbreviation: HPB Surg
NLM Journal ID: 9002972

HPB Surgery

ISSN: 0894-8569
EISSN: 1607-8462
Publisher: Wiley

ARTICLE INFORMATION
==============================
PMCID: PMC2423497
PMID: 3153778
DOI: 10.1155/1988/16253
Article ID: S089485698816253X
Article version: 1
Subjects: Research Article

Shunt Surgery Versus Endoscopic Sclerotherapy for Long-Term Treatment of Variceal Bleeding—Early Results of a Randomized Trial

Terblanche John
Dept. of surgery Univ. of Cape Town Medical School Cape Town South Africa
Print publication date: 1988
Volume: 1
Issue: 1
First page: 85
Last page: 89
Copyright: Copyright © 1988 Hindawi Publishing Corporation.
Copyright year: 1988
Copyright holder: Hindawi Publishing Corporation
License: This is an open access article distributed under the Creative Commons Attribution License, which permits unrestricted use, distribution, and reproduction in any medium, provided the original work is properly cited.
License URL: http://creativecommons.org/licenses/by/

==============================
*HPB Surgery* 1988, Vol. 1, pp. 85–89
Reprints available directly from the publisher
Photocopying permitted by license only

© 1988 Harwood Academic Publishers GmbH
Printed in Great Britain

### HPB INTERNATIONAL

### EDITORIAL & ABSTRACTING SERVICE

JOHN TERBLANCHE, EDITOR
Department of Surgery,
Medical School · Observatory 7925
Cape Town · South Africa
Telephone: Department Office: 47-1250
Hospital Connection: 47-3311
Telex: 5-22208
Tel. Add: ALUMNI CAPETOWN
Telefax: (021) 47-8955

As an additional service to our readers we have developed a section with the aim to review and comment on important work in the area of HPB and provide it with commentaries by leading scientists in the field. It is our hope that this will increase the value of our journal. We are happy to inform our readers that Professor John Terblanche has kindly accepted to be Editor of this section. John Terblanche will, through his extensive reading, identify important contributions and invite experts around the world to comment on them. We hope that readers who identify significant contributions in journals will send copies of the work to Professor Terblanche with the suggestion that the work might be reviewed and discussed. It is our hope that this cooperation will be successful. The Editor strongly desires to have a lively journal with active participation by its readers.

Stig Bengmark

### SHUNT SURGERY VERSUS ENDOSCOPIC SCLEROTHERAPY FOR LONG-TERM TREATMENT OF VARICEAL BLEEDING
### Early Results of a Randomized Trial

*Rikkers, L.F., Burnett, D.A., Volentine, G.D., Buchi, K.N. and Cormier R.A.* (1987) Annals of Surgery, 206, 261–271.

ABSTRACT

**In September 1982, a prospective randomized trial comparing shunt surgery and endoscopic sclerotherapy for the elective management of variceal hemorrhage in patients with cirrhosis was initiated. Twenty-seven patients have received shunts (distal splenorenal = 23, nonselective = 4) and 30 patients have had chronic sclerotherapy. Eighty-six per cent of patients had alcoholic cirrhosis and 33% were Child's class C. After a mean follow-up of 25 months, 19% of shunt and 57% of sclerotherapy patients have had rebleeding (p = 0.003). Kaplan-Meier survival**

**analysis reveals similar 2-year survival rates for shunt (65%) and sclerotherapy (61%) groups. Only two of 10 sclerotherapy failures have been salvaged by surgery. Post-therapy quantitative hepatic function, frequency of encephalopathy, and cumulative medical costs were similar for both groups. Hepatic portal perfusion and portal pressure at 1 year were better maintained by sclerotherapy than by distal splenorenal shunt. In conclusion, endoscopic sclerotherapy and shunt surgery provide similar results with respect to survival, hepatic function, frequency of encephalopathy, and costs. Sclerotherapy is an acceptable, but not superior, alternative to shunt surgery for treatment of variceal hemorrhage.**

### PAPER DISCUSSION

**Keywords:** Portacaval shunt; sclerotherapy, varices.

Repeated sclerotherapy is currently the most widely practised therapeutic measure in the long-term management of patients after a variceal bleed. It effectively eradicates oesophageal varices in most patients and after eradication recurrent variceal bleeding is significantly reduced[1–5]. Improvement in survival remains unproven particularly if the best available therapy, including sclerotherapy, is used for acute variceal bleeds when they occur[6]. Although repeated sclerotherapy is usually performed on an outpatient basis using a flexible endoscope without an anaesthetic, it is not without problems. Life long follow-up with repeated injections are required and complications become cumulative with time. Those patients who survive for prolonged periods place a major load on the hospital's resources.

Thus other forms of long-term management are continually under review. The most commonly used alternative procedure today is a porto-systemic shunt with the selective distal splenorenal shunt being the most popular. A successful shunt effectively prevents recurrent variceal bleeding but is unfortunately associated with unpredictable morbidity in the form of hepatic encephalopathy as well as mortality. The selective distal splenorenal shunt is favoured but is also not without problems. Of the six completed controlled trials[7–12] comparing the distal splenorenal shunt with conventional shunts only three have demonstrated a lesser immediate encephalopathy rate[8,9,12]. Although the maintenance of prograde portal vein flow is one of the theoretical advantages of a Warren shunt, which should lead to better preservation of liver function, not all patients with a Warren shunt maintain prograde portal perfusion[11,13]. This applies particularly to patients with alcoholic cirrhosis. Further problems with this shunt include its not being applicable to all patients especially those with marked ascites which is difficult to control and patients in poor general condition. Also survival in alcoholic cirrhosis is worse than in non-alcoholic cirrhosis[14]. These problems and reservations regarding both sclerotherapy and the popular distal splenorenal shunt make it vital that controlled trials be conducted.

The controlled trial under review undertaken by Rikkers *et al* demonstrated no difference between shunts and sclerotherapy. Because of the small number of patients included a type II error may have occurred. Thus, although no difference was found, with a greater number of patients and longer follow-up a difference might have been demonstrated.

Two other published controlled trials compared sclerotherapy with the distal splenorenal shunt. The first, from Warren's group at Emory, showed significantly improved survival in patients receiving sclerotherapy, when backed up by distal splenorenal shunt for the sclerotherapy failures (31% of patients)[15]. The patients undergoing sclerotherapy had a higher rebleed rate than those undergoing a distal splenorenal shunt while, on the other hand, sclerotherapy was associated with significantly improved liver function compared with shunts. The third trial showed no difference in survival between the sclerosed and shunted patients[16]. This group concluded that endoscopic sclerotherapy was a good alternative to splenorenal shunting in the elective treatment of oesophageal variceal bleeding, especially in patients prone to develop hepatic encephalopathy.

Once again, as with so many portal hypertension studies, controlled trials have resulted in conflicting results. The differences in these trials require further evaluation. There were subtle differences in the design of all three trials as well as variations in technique and in the types of patients included. Comparing Warren's and Rikkers' trial, Rikkers was unable to salvage the failures of sclerotherapy by shunt surgery. Four patients had salvage surgery and only two survived. The main difference in survival was that there was a higher survival rate for the sclerosed patients (including the shunt salvage patients) in the Emory trial[15] whereas the shunted patients in both groups had a similar survival. Sclerosed patients tended to die of bleeding while shunted patients died of liver failure. An important finding in both studies was that rebleeds after sclerotherapy were predominantly from gastric varices or portal hypertensive gastropathy. Such patients could probably only be salvaged by shunting and the early shunting of the sclerotherapy failures in Warren *et al's* trial was probably an important factor.

The Barcelona trial of Teres *et al*[16] contained a greater number of patients (112 patients), the sclerotherapy technique used was different including the use of ethanolamine oleate as sclerosant, and the distal splenorenal shunt was modified by performing it through a retroperitoneal approach without porto-azygous disconnection. This modification has been correctly criticised as not being a true distal splenorenal shunt. Despite careful analysis the reasons for the differences in outcome in these three trials remain obscure. Rikkers contends that somewhat better risk patients were included in the Warren study[15]. All the papers suggest that sclerotherapy is better than shunting in poor risk patients. The author believes that a reasonable approach would be to use repeated sclerotherapy as the first line treatment in all patients, but that any evidence of failure of sclerotherapy mandates a more radical surgical approach. The trials under discussion only considered shunting particularly the distal splenorenal shunt. Other trials are underway (including one in Cape Town) comparing sclerotherapy with extensive devascularisation and transection operations. When completed, they may indicate subsets of patients in whom these procedures may be preferable to shunts.

The trials under discussion all considered long-term therapy only and did not address the role of shunts or sclerotherapy in emergency therapy. In a controlled trial of emergency therapy in a selected group of poor risk patients Cello *et al*[17] have concluded that, although sclerotherapy is as good as end to side portacaval shunting in the acute management of variceal haemorrhage, sclerotherapy patients in whom varices are not obliterated and bleeding continues should be considered for elective shunt surgery. The costs and long-term survival did not differ significantly in the two groups.

Clearly the bottom line has yet to be written. Further carefully conducted controlled trials are essential in this important and difficult area. Finally, present evidence indicates that liver transplantation is a better therapeutic option than either sclerotherapy or shunts in poor risk patients, particularly those with non-alcoholic cirrhosis. This was emphasied by Iwatsuki in the discussion of the paper under review.

John Terblanche
Dept. of Surgery
Univ. of Cape Town Medical School
Cape Town, South Africa

### REFERENCES

1. Terblanche, J., Bornman, P.C., Kahn, D., Jonker, M.A.T., Campbell J.A.H., Wright, J. and Kirsch, R. (1983) Failure of repeated injection sclerotherapy to improve long-term survival after oesophageal variceal bleeding. A five year prospective controlled clinical trial. *Lancet*, ii, 1328–1332
2. The Copenhagen Esophageal Varices and Sclerotherapy Project. (1984) Sclerotherapy after first varical hemorrhage in cirrhosis. A randomized multicenter trial. *New England Journal of Medicine*, **311**, 1594–1600
3. Westaby, D., Macdougall, B.R.D. and Williams, R. (1985) Improved survival following injection sclerotherapy for esophageal varices. Final analysis of a controlled trial. *Hepatology*, **5**, 827–830
4. Korula, J., Balart, L.A., Radvan, G., Zweiban, B.C., Larson, A.W., Kao, H.W. and Yamada, S. (1985). A prospective, randomized controlled trial of chronic esophageal variceal sclerotherapy. *Hepatology*, **5**, 584–589
5. Soderlund, C. and Ihre, C. (1985) Endoscopic sclerotherapy *vs* conservative management of bleeding oesophageal varices. A 5-year prospective controlled trial of emergency and long-term treatment. *Acta Chirurgica Scandinavica*, **151**, 449–456
6. Terblanche, J. (1985) The long-term management of patients after an oesophageal variceal bleed: the role of sclerotherapy. *British Journal of Surgery*, **72**, 88–90
7. Harley, H.A.J. Morgan, T., Redeker, A.G., Reynolds, T.B., Villamil, F., Weiner, J.M. and Yellin, A. (1986) Results of a randomized trial of end-to-side portacaval shunt and distal splenorenal shunt in alcoholic liver disease and variceal bleeding. *Gastroenterology*, **91**, 802–809
8. Millikan, W.J., Warren, W.D., Henderson, J.M., Smith, R.B., Salam, A.A., Galambos, J.T., Kutner, M.H. and Keen, J.H. (1985) The Emory prospective randomized trial: selective versus nonselective shunt to control variceal bleeding. Ten-year follow-up. *Annals of Surgery*, **201**, 712–722
9. Langer, B., Taylor, B.R., Mackenzie, D.R., Gilas, T., Stone, R.M. and Blendis, L. (1985) Further report of a prospective randomized trial comparing distal splenorenal shunt with end-to-side portacaval shunt. An analysis of encephalopathy, survival, and quality of life. *Gastroenterology*, **88**, 424–429
10. Conn, H.O., Resnick, R.H., Grace, N.D., *et al.* (1981) Distal splenorenal shunt *vs* portal-systemic shunt: current status of a controlled trial. *Hepatology*, **1**, 151–160
11. Fisher, J.E., Bower, R.H., Atamian, S. and Welling, R. (1981) Comparison of distal and proximal splenorenal shunts. A randomized prospective trial. *Annals of Surgery*, **194**, 531–544
12. Reichle, F.A., Fahmy, W.F. and Golsorkhi, M. (1979) Prospective comparative clinical trial with distal splenorenal and mescaval shunts. *American Journal of Surgery*, **137**, 13–21
13. Maillard, J.N., Flemant, Y.M., Hay, J.M. and Chandler, J.G. (1979) Selectivity of the distal splenorenal shunt. *Surgery*, **86**, 663–71
14. Henderson, J.M., Millikan, W.J., Wright-Bacon, L., Kutner, M.H. and Warren, W.D. (1983) Hemodynamic differences between alcoholic and non-alcoholic cirrhotics following distal splenorenal shunt - effect on survival? *Annals of Surgery*, **198**, 325–334
15. Warren, W.D., Henderson, J.M., Millikan, W.J., Galambos, J.T., Brooks, W.S., Riepe, S.P., Salam, A.A. and Kutner, M.H. (1986) Distal splenorenal shunt versus endoscopic sclerotherapy for long-term management of variceal bleeding. Preliminary report of a prospective, randomized trial. *Annals of Surgery*, **203**, 454–462
16. Teres, J., Bordas, J.M., Bravo, D., Visa, J., Grande, L., Garcia-Valdecasas, J.C., Pera, C. and Rodes, J. (1987) Sclerotherapy vs. distal splenorenal shunt in the elective treatment of variceal hemorrhage: a randomized controlled trial. *Hepatology*, **7**, 430–436
17. Cello, J.P., Grendell, J.H., Crass, R.A., Weber, T.E. and Trunkey, D.D. (1987) Endoscopic sclerotherapy versus portacaval shunt in patients with severe cirrhosis and acute variceal hemorrhage. Long-term follow-up. *New England Journal of Medicine*, **316**, 11–15.

### SELECTED HPB INTERNATIONAL

### *INSTRUCTIONS TO AUTHORS*

This section aims to provide the readers of *HPB Surgery* with a selected abstracting service with a difference. The Editor, the Editorial board of *HPB Surgery* and local colleagues will identify and select important and seminal papers in the field of hepatic, pancreatic and biliary surgery published in other major journals. Readers are also encouraged to contact the Editor of this section with suggestions on papers to be included. An authority in the field will be asked to comment in a signed short essay or editorial on the selected papers. Both the original abstract (or summary of the abstract) and the expert essay will be published.

The essay should be approximately two printed page (four to five double type-written pages) and contain a few selected references. It is hoped that the experts will usually applaude but sometimes criticise the paper and will add relevant comment. The format will be left to the individual essayist.

The original authors will not be asked to comment on the essay, but are invited to submit comments, if they wish, for a special section entitled "Selected HPB International Correspondence". Here they will be afforded early publication. The Editor believes that in most instances there will be no need for the authors to reply.

The essay must conform to the style of *HPB Surgery* (see instructions to authors).

Readers comments on the aims and format of the Selected HPB International will be welcomed by the section editor.
